# Emergence of ST11-K47 and ST11-K64 hypervirulent carbapenem-resistant *Klebsiella pneumoniae* in bacterial liver abscesses from China: a molecular, biological, and epidemiological study

**DOI:** 10.1080/22221751.2020.1721334

**Published:** 2020-02-09

**Authors:** Qiwen Yang, Xinmiao Jia, Menglan Zhou, Hui Zhang, Wenhang Yang, Timothy Kudinha, Yingchun Xu

**Affiliations:** a Department of Clinical Laboratory, Peking Union Medical College Hospital, Peking Union Medical College, Chinese Academy of Medical Sciences, Beijing, People’s Republic of China; b Beijing Key Laboratory for Mechanisms Research and Precision Diagnosis of Invasive Fungal Diseases, Beijing, People’s Republic of China; c Central Research Laboratory, Peking Union Medical College Hospital, Peking Union Medical College, Chinese Academy of Medical Sciences, Beijing, People’s Republic of China; d Graduate School, Peking Union Medical College, Chinese Academy of Medical Sciences, Beijing, People’s Republic of China; e School of Biomedical Sciences, Charles Sturt University, Orange, Australia; f Pathology West, NSW Health Pathology, Orange, Australia

**Keywords:** Hypervirulent, carbapenem-resistant, Klebsiella pneumoniae, bacterial liver abscesses

## Abstract

**Background:** Multidrug-resistant bacteria, especially those with high virulence, are an emerging problem in clinical settings.

**Methods:** We conducted a multicentre epidemiological and comparative genomic analysis on the evolution, virulence and antimicrobial resistance of carbapenem-resistant *Enterobacteriaceae* in patients with bacterial liver abscesses from 2012 to 2016.

**Results:** A total of 477 bacterial isolates were collected. *Enterobacteriaceae* were the main pathogen (89.3%) with *K. pneumoniae* (52.4%) predominating followed by *Escherichia coli* (26.8%). All CRKps (3.2%) were of sequence type (ST) 11 and serotypes K47 or K64, and simultaneously possessed acquired *bla*_KPC-2_/*bla*_KPC-5_ and *bla*_CTX-M-65_ together with the multidrug transporter EmrE. Seven Hv-CRKps (five ST11-K47, two ST11-K64) were confirmed by bacteriological test, neutrophil killing assay and *Galleria mellonella* infection model. Genomic analysis indicated that the emergence of one ST11-K64 Hv-CRKp strain was related to the acquisition of *rmpA/rmpA2* genes and siderophore gene clusters, while ST11-K47 Hv-CRKp lacked these traditional virulence genes. Further complete genome analysis of one ST11-K47 Hv-CRKp strain, R16, showed that it acquired a rare plasmid (pR16-Hv-CRKp1) carrying *bla*_KPC-2_, *bla*_SHV-12_, *bla*_TEM-1_, *bla*_CTX-M-65_, *rmtB* and a predicted virulence gene R16_5486 simultaneously.

**Conclusion:** The emergence of the ST11-K47/K64 Hv-CRKps, which are simultaneously multidrug-resistant and hypervirulent, requires urgent control measures to be implemented.

## Introduction

The increased prevalence of antibiotic resistance among pathogens is nearly at critical levels. In 2016, the World Health Organization (WHO) came up with a pathogen list of the most important resistant bacteria at a global level for which there is an urgent need for new treatments, with carbapenem-resistant *Enterobacteriaceae* (CRE) classified as a critical priority organism. Infections caused by CRE have emerged worldwide, posing a global public health threat and a formidable challenge to antimicrobial therapy [1,2]. Among these, carbapenem-resistant *Klebsiella pneumoniae* strains (CRKp) account for roughly 70–90% of clinical CRE infections in the European Union and China [3,4].

Bacterial liver abscess is a relatively common infection caused by a wide variety of bacteria. Before mid-1980s, *Escherichia coli* was the most common etiological agent of pyogenic liver abscesses (PLA), until a new hypervirulent variant of *K. pneumoniae* (HvKp) causing invasive PLA was firstly described in Taiwan, and subsequently found worldwide, especially in Asia, with the most common serotypes being K1 and K2 [5,6]. To date, HvKp is considered to surpass *E. coli* to become the predominant cause of PLA over the past three decades [7]. Although most of the HvKp strains identified to date are susceptible to most antimicrobials, some cases of multidrug-resistant HvKp infections have been reported [8]. Most recently, carbapenem-resistant HvKp (CR-HvKp) isolated from different clinical settings has begun to emerge in China [9,10]. The possibility of enhanced virulence and acquisition of carbapenem resistance in HvKp strains further complicates clinical practice.

Despite the low incidence of Hv-CRKp strains in China, few studies have been performed to investigate the epidemiology of bacterial liver abscesses caused by CRE. In this study, we conducted a multi-centre study to understand the pathogen epidemiology of bacterial liver abscess, and also carried out a comparative genomic analysis focusing on CR-HvKp strains, to better clarify the virulence and antimicrobial resistance mechanisms involved.

## Methods

### Bacterial strains

Gram-negative bacteria were consecutively collected from patients with clinical- and laboratory-confirmed bacterial liver abscesses, diagnosed based on the Johns Hopkins ABX Guide (https://www.hopkinsguides.com/hopkins/view/Johns_Hopkins_ABX_Guide/540259/all/Hepatic_Abscess) between 2012 and 2016 in 15 centres located in 11 Chinese cities. All organisms were considered clinically significant by local hospital criteria and were isolated from high-quality specimens, including liver tissues collected in surgery or from liver abscess aspirates. All isolates were sent to the central clinical microbiology laboratory of Peking Union Medical College Hospital (PUMCH) for identification confirmation using MALDI-TOF MS (Vitek MS, BioMérieux, France). The Human Research Ethics Committee of PUMCH approved this study (Ethics Approval Number: S-K238).

### Antimicrobial susceptibility testing

Antimicrobial susceptibility testing was conducted by broth microdilution method as per the Clinical and Laboratory Standards Institute (CLSI) recommendations [11]. MICs were interpreted following the CLSI M100-S28 guidelines [11]. *E. coli* ATCC 25922, *Pseudomonas aeruginosa* ATCC 27853 and *K. pneumoniae* ATCC700603 were used as quality controls.

### Virulence assay using the string test, neutrophil killing assay, and *Galleria mellonella* infection model

To identify the hypermucoviscous phenotype of *K. pneumoniae*, string test was performed as previously described [12]. The result was deemed positive when a viscous string longer than 5 mm could be generated by touching and pulling a single colony upwards with a standard inoculation loop. Neutrophil killing assay (NKA) was also performed to test the virulence potential of *K. pneumoniae* isolates [13]. Furthermore, we tested the virulence potential of the isolates in *G. mellonella* larvae weighing approximately 300 mg (purchased from Tianjin Huiyude Biotech Company, Tianjin, China), as previously described. A known hypervirulent *Klebsiella pneumoniae* strain 1088, a gift from Dr. Zhang R was used as the positive control [9]. A classic *K. pneumoniae* strain QD110 identified in this study was added as a negative control (see detailed information in Material S3).

### Genomic DNA extraction, sequencing, assembly and annotation

To better understand the genetic basis of CRE strains, all CRE (study group) and 12 carbapenem-susceptible *Enterobacteriaceae* (CSE) (comparative group) were further analyzed for whole-genome sequencing. Single risk factor analysis concerning sex, age, patient source and some more detailed clinical information between CRE and CSE group was performed using fisher's exact test or Mann–Whitney test. Multivariate Logistic Regression analysis was not performed due to the limited sample sizes. TIANamp Bacteria Genomic DNA Kit (TiangenBiotechCo. Ltd., Beijing, China) and Illumina Genome Analyzer 2X technology (Illumina, San Diego, CA, USA) were used for the genomic DNA extraction and shotgun sequencing. Adapters and low-quality sequences were trimmed and filtered, and SPAdes v3.11was used for the de novo assembly of these reads [14]. Whole-genome sequencing of one ST11-K47 Hv-CRKp strain R16 was performed using Pacific Biosciences Sequel System (Pacific Biosciences, Menlo Park, CA, USA). The sequencing reads of other seven CRKps were mapped on the genome of R16 using BWA 0.5.9, and genome coverage, gene coverage and SNPs were analyzed using samtools and bedtools (see detailed information in Supplementary Material S3).

### Sequence types (STs) and serotype analysis

STs were determined based on multilocus sequence typing (MLST, http://bigsdb.Pasteur.fr/klebsiella/klebsiella.html, http://mlst.warwick.ac.uk/mlst/dbs/E.coli). It was also re-confirmed using SRST2 based on the Illumina reads [15]. Serotypes were identified based on the whole genome data using Kaptive (https://github.com/katholt/Kaptive) together with the Blast results between the known primer sequences and assembled scaffolds [16].

### Single nucleotide polymorphism (SNP) identification and phylogenetic analysis

SNPs were identified by MUMmer using the published complete genomes. Paired-end reads of *K. pneumoniae* were mapped to the HS11589 genome (NC_016845.1), and paired-end reads of *E. coli* were mapped to the O157:H7 genome (NC_002695.1) using BWA 0.5.9. SNPs were analyzed using SAMtools 0.1.19. SNPs located in repetitive regions of the reference genome, defined as exact repetitive sequences ≥25 bp in length as identified using RepeatMasker (http://www.repeatmasker.org/cgi-bin/WEBRepeatMasker), were excluded. Phylogenetic tree was constructed using the SNPs described in all strains based on the maximum likelihood in MEGA7 [17] (see detailed information in Material S3).

### Virulence genes, antimicrobial resistance genes and homologous genes analysis

Virulence genes in *K. pneumoniae* and *E. coli* were downloaded from the *K. pneumoniae* BIGSdb (http://bigsdb.Pasteur.fr/klebsiella/klebsiella.html) and Virulence Factor Database (VFDB), respectively. Antimicrobial resistance genes were downloaded from the files of ResFinder 3.0 in SRST2 [15]. The virulence and antimicrobial resistance genes in our sequenced strains were predicted using SRST2.

### Analysis of plasmids from R16

The plasmids of R16 were further annotated based on ExPASy and NCBI NR databases, and the closely related plasmids [18]. The genome structure comparisons among plasmids were performed to analyze the sequence homology. Plasmid circular structure maps were generated with BRIG software. Prophage analysis was conducted by PHAST [19].

## Results

### Demographic information and pathogen distribution of patients with bacterial liver abscesses

**Figure 1.**
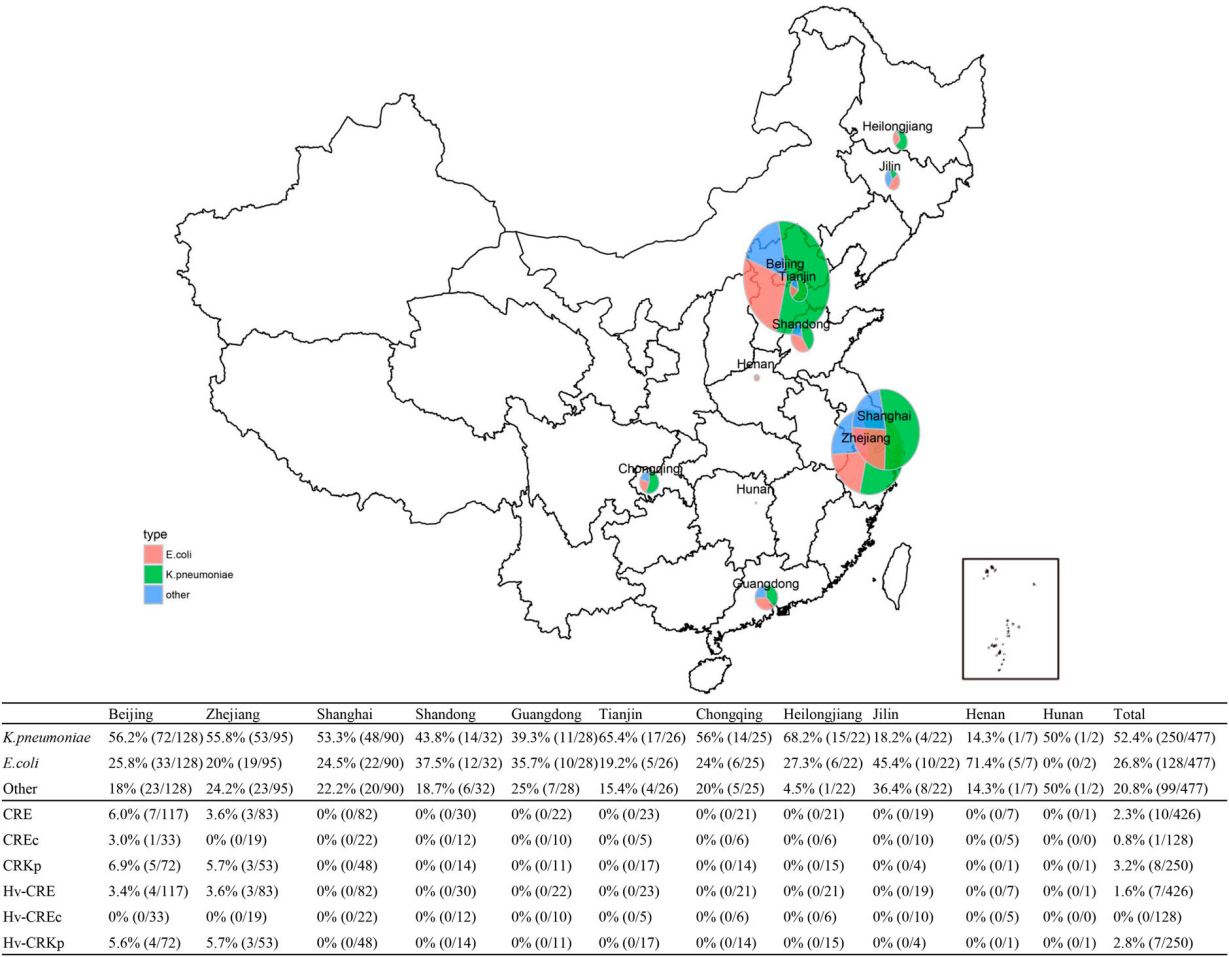
Geographic distribution of isolates obtained from bacterial liver abscesses from 2012 to 2016, China.

A total of 477 Gram-negative bacteria isolated from 477 patients with clinical- and laboratory-confirmed bacterial liver abscesses, were studied (Figure 1). Two-hundred and ninety-one patients (61.0%) were males; the mean age was 59.9 ± 13.1 years. *Enterobacteriaceae* was the main family of pathogens isolated (426, 89.3%), with *K. pneumoniae* (250, 52.4%) and *E. coli* (128, 26.8%) being the top two most commonly isolated species. Non-fermenting Gram-negative bacteria comprised 10.3% of the observed pathogens, with *A. baumannii* (24, 5.0%) and *P. aeruginosa* (19, 4.0%) being the two most common (Table 1).

**Table 1.** The demographic information and pathogen distribution of patients who presented with bacterial liver abscesses from 2012 to 2016, China.

Gender	Male (*N* = 291, 61.0%)
	Female (*N* = 186, 39.0%)
Age (years)	59.9 ± 13.1
Pathogen distribution [*N*(percentage)]	
Enterobacteriaceae	426 (89.3%)
*Klebsiella pneumoniae*	250 (52.4%)
*Escherichia coli*	128 (26.8%)
*Enterobacter cloacae*	12 (2.5%)
*Citrobacter freundii*	9 (1.9%)
*Proteus mirabilis*	7 (1.5%)
*Enterobacter aerogenes*	6 (1.3%)
*Klebsiellaoxytoca*	5 (1.0%)
*Serratia marcescens*	2 (0.4%)
*Citrobacter amalonaticus*	2 (0.4%)
*Citrobacter koseri*	1 (0.2%)
*Klebsiella ozaenae*	1 (0.2%)
*Enterobacter asburiae*	1 (0.2%)
*Morganella morganii ss. morganii*	1 (0.2%)
*Proteus vulgaris*	1 (0.2%)
Non-fermenting gram-negative bacteria	49 (10.3%)
*Acinetobacter baumannii*	24 (5.0%)
*Pseudomonas aeruginosa*	19 (4.0%)
*Stenotrophomonas maltophilia*	5 (1.0%)
*Acinetobacter* spp.	1 (0.2%)
Other	2 (0.4%)
*Aeromonas hydrophila*	1 (0.2%)
*Aeromonas* spp*.*	1 (0.2%)

### Antimicrobial susceptibility of isolates obtained from bacterial liver abscesses showing the emergence of CRE

Eleven antibiotics were used for antimicrobial susceptibility testing of 426 *Enterobacteriaceae* isolates. A total of ten (2.4%) CRE (resistant to ertapenem or imipenem) were detected (Figure 1). *K. pneumoniae* isolates exhibited a high level of susceptibility to all antimicrobial agents tested, and were most susceptible to ertapenem, imipenem, amikacin, and piperacillin-tazobactam. CRKp accounted for 3.2% (8/250) of all the *K. pneumoniae* isolates (Table 2). Similarly, *E. coli* isolates also showed a high level of susceptibility to imipenem, ertapenem and amikacin (>90%) with CREc accounting for 0.8% (1/128) (Table 2). Moreover, CRKps and CREcs only occured in Beijing and Zhejiang (Figure 1). Apart from *Enterobacteriaceae*, *A. baumannii* showed a high level of resistance to all antimicrobial agents tested with susceptibility rates of <40% (Table 2).

**Table 2.** Antimicrobial susceptibility of *K. pneumoniae*, *E. coli* and *A. baumannii* isolates obtained from bacterial liver abscesses from 2012 to 2016, China.

Organisms/Antimicrobial agents	%R	%I	%S	MIC_50_	MIC_90_
*K. pneumoniae* (*n* = 250)					
Piperacillin/Tazobactam	4.8	3.6	91.6	2	16
Ceftazidime	10.8	0.8	88.4	0.5	16
Ceftriaxone	16.7	0.8	82.5	1	64
Cefotaxime	18.3	0.4	81.3	0.5	64
Cefepime	13.1	2	84.9	0.5	32
Cefoxitin	11.6	6	82.5	4	32
Ertapenem	3.2	1.6	95.2	0.032	0.125
Imipenem	3.2	1.2	95.6	0.5	1
Amikacin	3.6	0	96.4	4	4
Ciprofloxacin	13.5	2.4	84.1	0.25	4
Levofloxacin	12	1.6	86.5	0.5	8
*E. coli* (*n* = 128)					
Piperacillin/Tazobactam	10.2	3.9	85.9	2	128
Ceftazidime	40.6	8.6	50.8	4	64
Ceftriaxone	71.9	1.6	26.6	64	64
Cefotaxime	72.7	0	27.3	64	256
Cefepime	60.2	6.2	33.6	32	64
Cefoxitin	22.7	9.4	68	8	32
Ertapenem	0.8	5.5	93.8	0.032	0.5
Imipenem	0	0.8	99.2	0.125	0.5
Amikacin	7	0.8	92.2	4	8
Ciprofloxacin	62.5	1.6	35.9	4	4
Levofloxacin	50.8	9.4	39.8	8	8
*A. baumannii* (*n* = 24)					
Piperacillin/Tazobactam	66.7	4.2	29.2	128	128
Ceftazidime	66.7	8.3	25	64	256
Ceftriaxone	70.8	4.2	25	64	64
Cefotaxime	70.8	8.3	20.8	64	256
Cefepime	70.8	0	29.2	64	64
Imipenem	66.7	8.3	25	16	32
Amikacin	58.3	4.2	37.5	64	64
Ciprofloxacin	66.7	0	33.3	4	4
Levofloxacin	50	16.7	33.3	4	8

### Clinical information and genetic characteristics of CRE and CSE

CRE (8 *K. pneumoniae*, 1 *E. coli* and 1 *E. cloacae*) were isolated from ten patients aged between 23 and 67 years old in five different hospitals. Four of them had an in-patient history and/or broad-spectrum antibiotic usage within 90 days before infection. They all underwent surgery intervention and at least five days’ treatment with various antibiotics. Carbapenems were used in three of the ten patients, among which the two treated with ertapenem with or without tigecycline got improved and discharged; the one treated with meropenem was not improved based on clinical and bacteriological assessment. Clinical and bacteriological assessment revealed six remission cases with assumed pathogen clearance, four inefficiency or deterioration with two assumed pathogen non-clearance and two pathogens persisting. Nine patients (six improved and three didn't improve) were discharged from hospital after treatment and the remaining one died from infection after 20 days of treatment (Supplementary Table S4).

CSE (7 *K. pneumoniae* and 5 *E. coli*) were isolated from ten patients aged between 39 and 67 years old in eight different hospitals. Five of them had an in-patient history and/or broad-spectrum antibiotic usage within 90 days before infection. Imipenem was administered in ten of the patients during their treatment progress. All the patients underwent surgery intervention procedures. Clinical and bacteriological assessment revealed 11 remission cases with eight assumed pathogen clearance, one with assumed not clearance and two with pathogen persisting; one inefficiency or deterioration with pathogen persisting. All but one patient got improved and discharged from hospital (Supplementary Table S4).

Risk factor analysis concerning sex, age, patient source and some more detailed clinical information between CRE and CSE group was performed but none of these factors were considered statistically significant (*p *> 0.05) (Supplementary Table S5).

### MLST genotyping, serotypes and phylogenetic analysis of CRE strains

**Figure 2.**
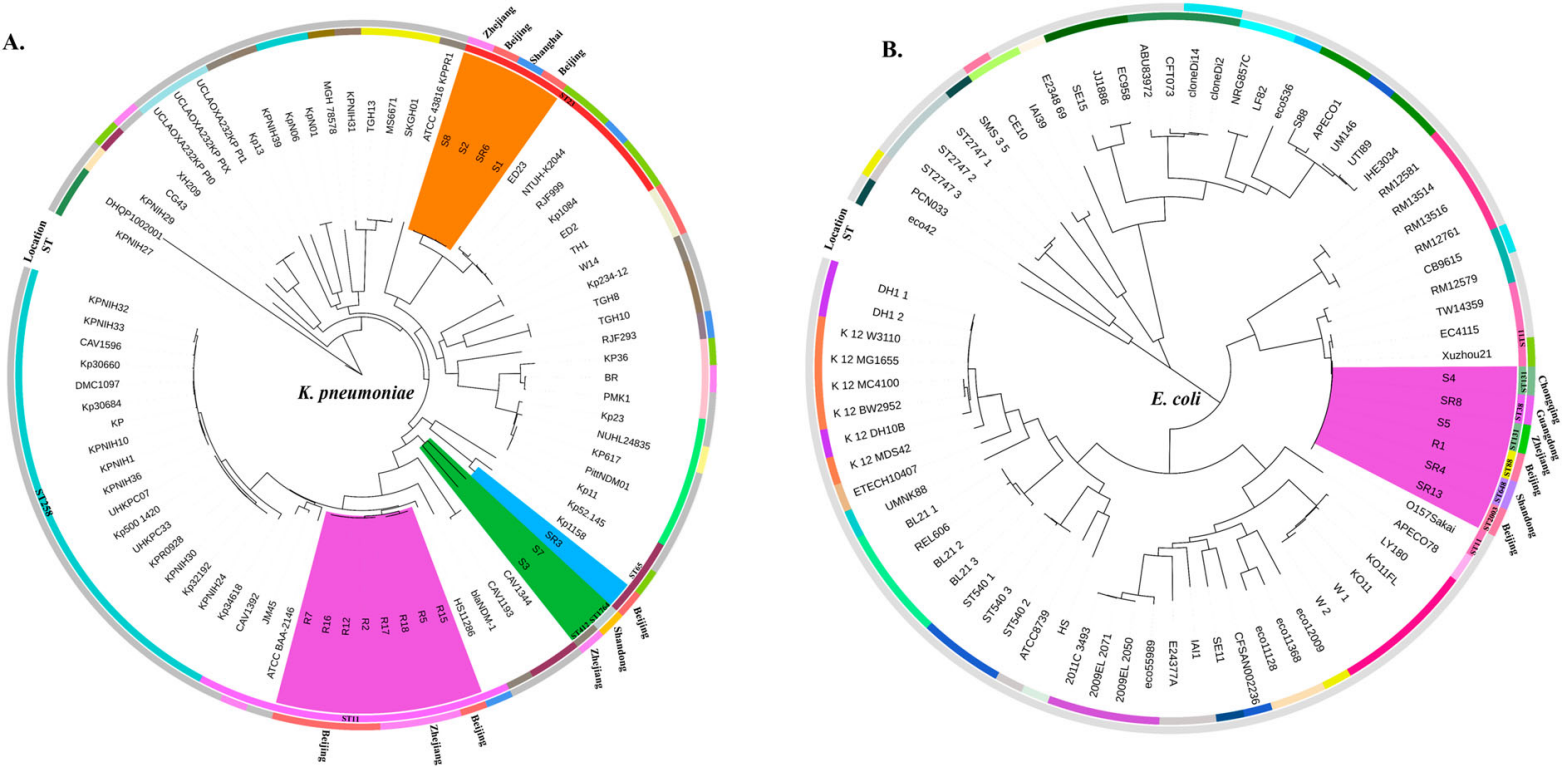
Phylogenetic analysis of *K. pneumoniae* (A) and *E. coli* (B) strains. Fifteen *K. pneumoniae* strains in this study are highlighted in different colours as follows: carmine, ST11; orange, ST23; green, ST412 and ST1764; and blue, ST65. Six *E. coli* strains in this study are highlighted in carmine. Circles outside the tree indicate the STs and locations of each strain. Different STs and locations are marked with different colours, and strains not from China are coloured grey.

To better understand the genetic basis of CRE strains, the 10 CRE (8 *K. pneumoniae*, 1 *E. coli* and 1 *E. cloacae*) and 12 CSE (7 *K. pneumoniae* and 5 *E. coli*), isolated from the same geographic area and same collection period as the 10 CRE, were further analyzed. MLST and serotype results showed that all the CRKps were ST11 and serotypes K47 and K64, whilst most of the CSKps strains belonged to ST23 and serotype K1. We also detected one rare ST1764 CSKp strain. In contrast to the CRE, the CSEcs were of diverse STs (Figure 2), and one CREc strain belonged to ST88. Moreover, 64 *K. pneumoniae* and 70 *E. coli* strains with complete genomes were downloaded from NCBI for phylogenetic tree construction based on whole-genome SNPs (Table S1). As shown in Figure 2, all CRKps of ST11 were clustered together with ST11 strain HS11286 (NC_016845). ST11-K47 and ST11-K64 strains appear to have evolved separately into two different branches. The four ST23 CSKps were phylogenetically closely related to the K1 hypervirulent strain NTUH-K2044 (NC_012731). The other three CSKp strains clustered with the K2 hypervirulent strain Kp52.145 (NZ_FO834906).

Regarding *E. coli*, the one CREc and five CSEc strains were clustered in one branch with O157Sakai (Japan 1996 outbreak isolate) and Xuzhou21 (a novel *E.coli* O157:H7 clone that caused a major hemolytic uremic syndrome outbreak in China) [20]. These results indicate a close relationship among these strains.

### Comparative analysis of antibiotic resistance genes

**Figure 3.**
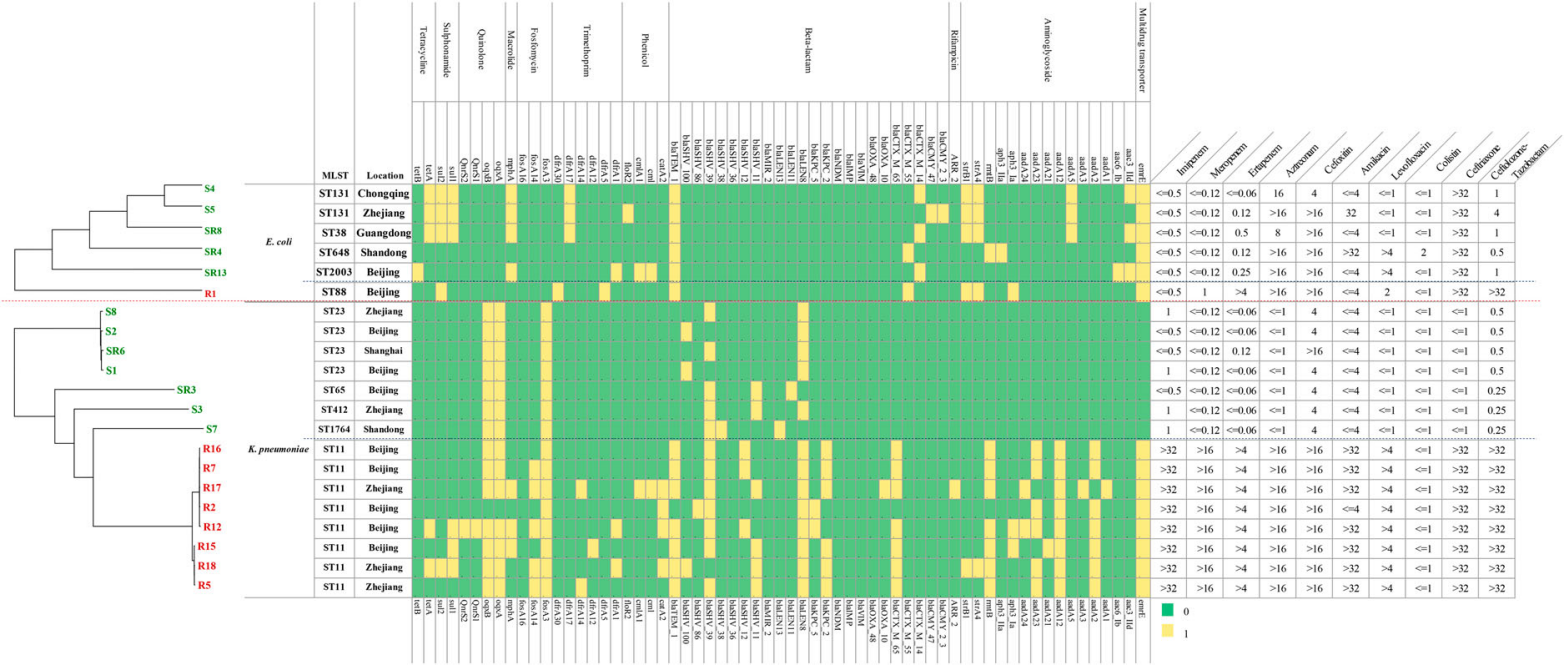
Evolutionary relationships, antibiotic resistance genes, distribution and MIC values of *K. pneumoniae* and *E. coli* strains. Evolutionary relationships, antimicrobial resistance genes and MICs are shown on the left, middle and right, respectively. Strains are colour coded on the tree (CRE, red; CSE, green). Each row of the heatmap (middle) indicates a strain, and each column represents an antibiotic resistance gene that belongs to the indicated functional clusters shown at the top. The colour intensity shows the copy number of the antibiotic resistance gene.

Comparative genomic analysis of antibiotic resistance genes indicated that the CRE strains harboured more types and numbers of drug-resistance genes than those found in CSE. In addition, these CRKps contained almost all classes of genes conferring resistance to aminoglycoside, rifampicin, phenicol, trimethoprim, fosfomycin, macrolide, quinolone, sulphonamide, tetracycline and beta-lactam (Figure 3). Moreover, carbapenemase genes (*bla*_KPC-2_ or *bla*_KPC-5_) and extended spectrum *β*-lactamase (ESBL) genes (*bla*_CTX-M-65_) simultaneously existed in CRKp strains. Interestingly, multidrug transporter ErmE that were initially described in *E. coli*, have now been acquired by all CRKps but not CSKps (Figure 3). This gene expels positively charged hydrophobic drugs, thereby conferring resistance to a wide range of toxic compounds [21].

### Virulence phenotype and genetic characteristics of CRE indicate the emergence of ST11-K47 and ST11-K64 Hv-CRKps

#### Virulence phenotype characterization of CRE

**Figure 4.**
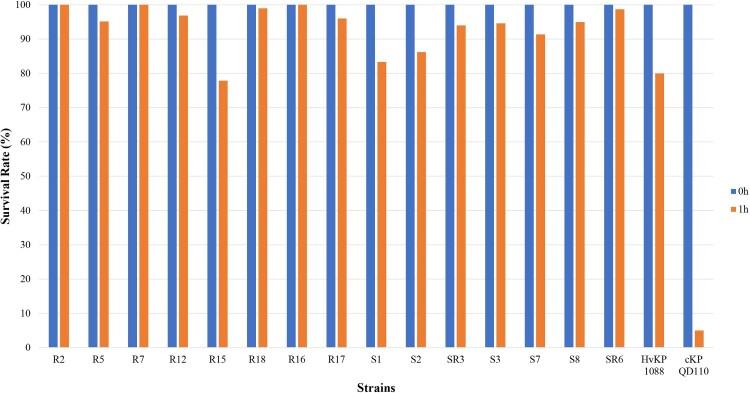
Human neutrophil assays of CRKp and CSKp.

**Figure 5.**
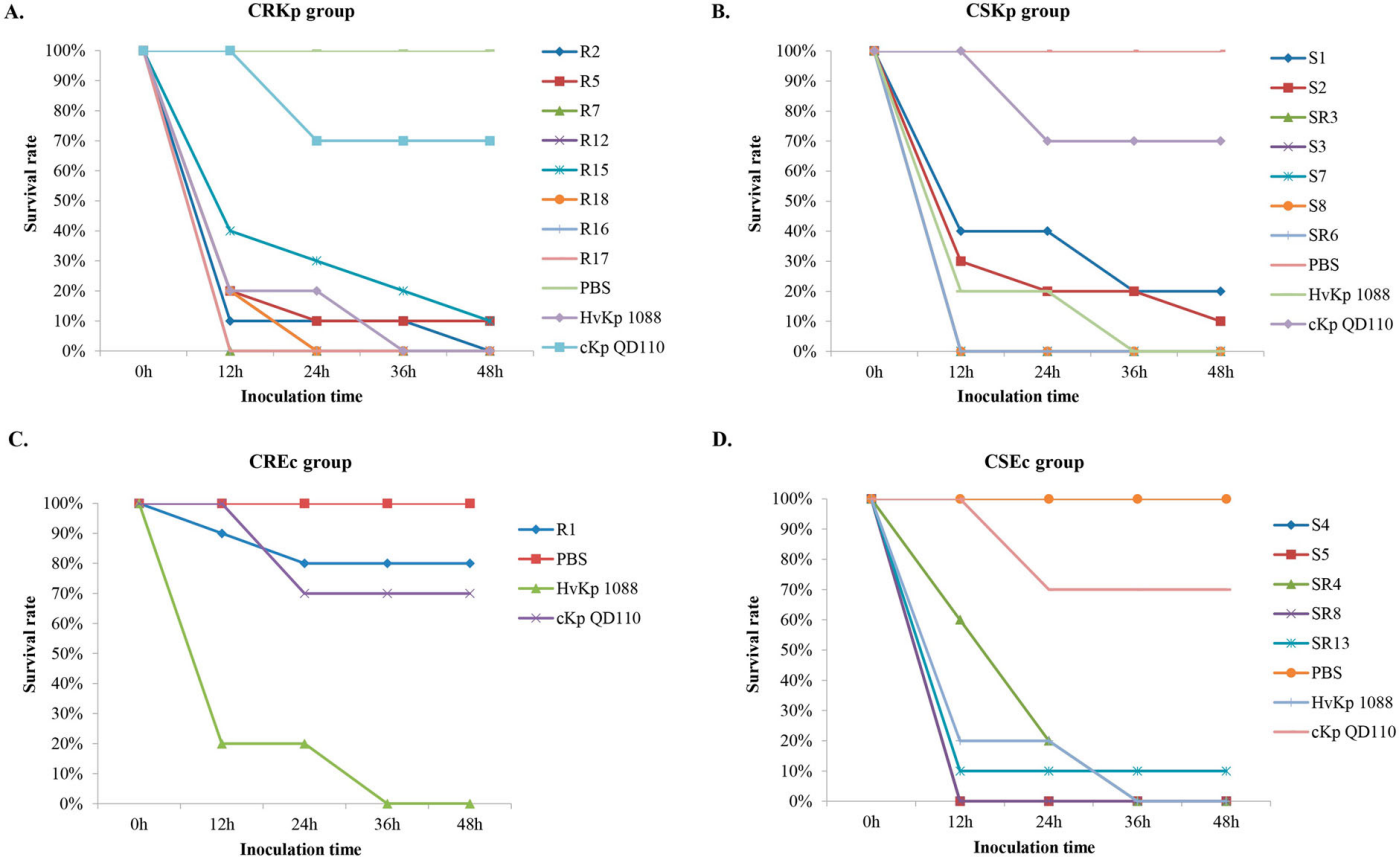
Virulence potential of *K. pneumoniae* and *E. coli* strains in a *G. mellonella* infection model. The effect of 1 × 10^6^CFU of each strain on survival was assessed in *G.mellonella*. (A) CRKp group. (B) CSKp group. (C) CREc group. (D) CSEc group.

A positive string test was observed in 0/8 of the CRKp and 4/7 of the CSKp isolates. Human NKA results showed that 7/8 of the CRKp and all the CSKp isolates exhibited higher survival rates than strain 1088 (80%) and thus classified as HV. Only one of the CRKp was considered to be MV with a survival rate of 77.9% (Figure 4). Further animal testing results using a *G. mellonella* infection model are shown in Figure 5. Generally, the CRKp and CREc isolates exhibited relatively lower virulence than CSKp and CSEc isolates. However, all five ST11 CRKps isolates with capsular serotype K47 and two ST11-K64 CRKp isolates showed high virulence in the CRKp group. Strains R16, R7 and R17 showed the highest virulence level, exhibiting a survival rate of 0% at 12 h with an inoculation of 10^6^ CFU of bacteria, followed by R2, R12, R18 and R5 (survival rates less than 20% at 12h), comparable to that of strain 1088. In the CSKp group, S7, S3, SR3, S8 and SR6 showed HV with a survival rate of 0% at 12h, most of which were ST23 belonging to the familiar capsular serotypes K1, K2 or K57. For *E. coli*, four CSEc isolates belonged to the HV group; the remaining one CSEc and one CREc isolates belonged to the low virulence group.

#### Comparative analysis of virulence genes

**Figure 6.**
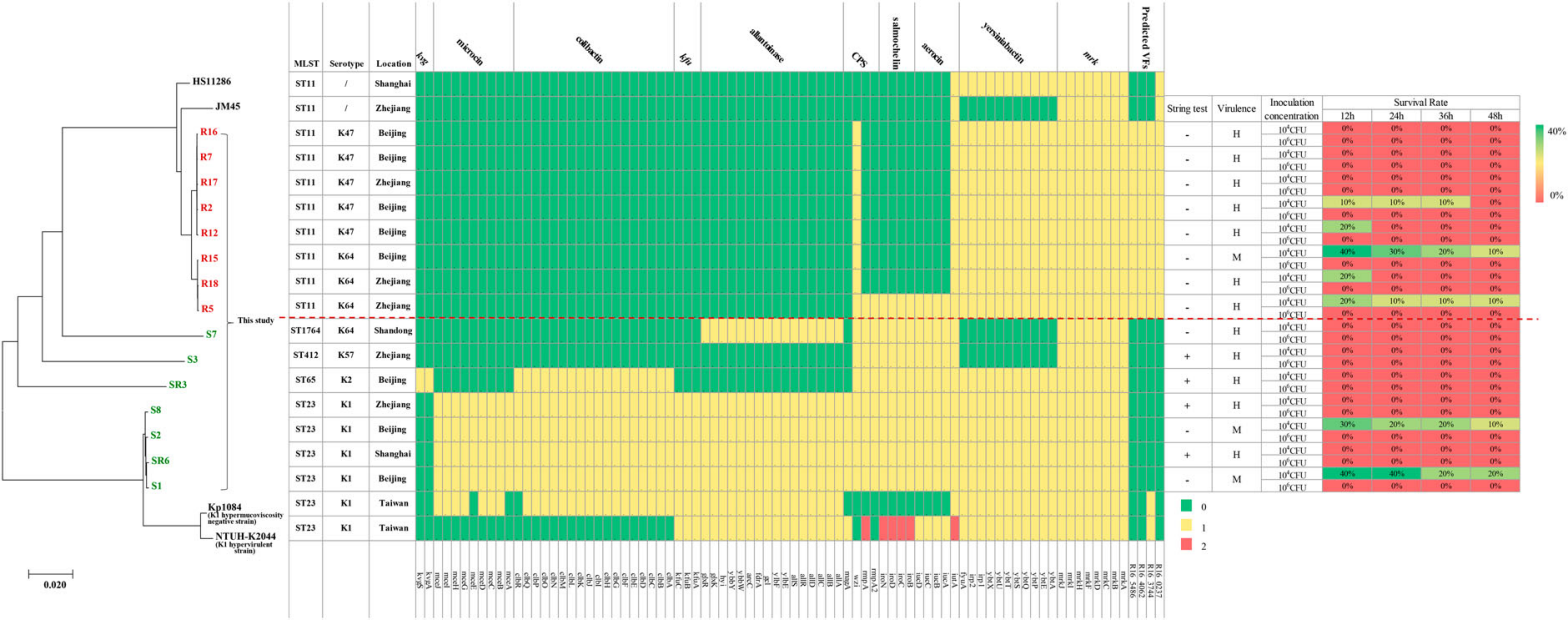
Virulence gene distribution and virulence assay of 15 *K. pneumoniae* strains in a *G. mellonella* infection model. Evolutionary relationships, virulence genes and virulence phenotypes are shown on the left, middle and right, respectively. Two K1 hypervirulent and two ST11 online complete genomes were selected from the NCBI GenBank as references. Our 15 strains are colour coded on the tree (CRKp, red; CSKp, green). Each row of the heatmap (middle) indicates a strain, and each column represents a virulence gene that belongs to the indicated 10 functional clusters shown at the top. The colour intensity shows the copy number of the virulence gene. The virulence assay was conducted using the *G. mellonella* infection model, and the survival rates of *G. mellonella* infected with the respective strains are shown on the right.

A total of 79 *K. pneumoniae* virulence genes were downloaded from the *K. pneumoniae* BIGSdb database, and 273 virulence genes of *E. coli* were downloaded from the VFDB. Comparative virulence gene analysis was conducted among these isolates (Figure 6 and Figure S1). As shown in Figure 6, fewer virulence genes were possessed by CRKps than CSKps generally. In the CSKp group, all Hv-CSKps isolates carried the capsular polysaccharide (CPS) regulator genes (*rmpA*/*rmpA2*), salmochelin and aerocin siderophore gene clusters, which have been reported to be related to the hypervirulence phenotype of *K. pneumoniae* [12]. In the CRKp group, only the ST11-K64 Hv-CRKp strain R5 acquired the CPS regulator genes and siderophore genes, which was consistent with its hypervirulence phenotype. The remaining two ST11-K64 and five ST11-K47 Hv-CRKp strains lacked these virulence genes, which implies the likely presence of new virulence genes that need to be discovered.

However, the *wzi* gene and another two virulence genes, yersinibactin and *mrk*, were observed in all the Hv-CRKp strains [22]. Therefore, we further conducted a homologous gene analysis against VFDB between the strains with different phenotypes. Four predicted virulence genes (R16_0237, R16_3744, R16_4062, R16_5486) were described only in CRKps compared with our seven CSKps (Figure 6). Moreover, the predicted virulence gene, R16_3744, was also found in Kp1084 and the hypervirulent strain NTUH-K2044, yet more work need to be done. In contrast, the analysis of virulence genes in *E. coli* revealed no obvious features (Figure S1 and Table S2).

### Complete genome and detailed plasmids analysis of the ST11-K47 Hv-CRKp strain R16

In order to gain further insights into the genetic basis of virulence and drug resistance of Hv-CRKp strains in this study, one ST11-K47 Hv-CRKp strain R16 was further sequenced using PacBio Sequel system. Through genome assembly, we obtained the first complete genome sequence of ST11-K47 Hv-CRKp strain. The bioinformatic analysis providing the general genome information is shown in Table 3.

**Table 3.** Genome information of ST11-K47 Hv-CRKp strain R16.

Name	Genome size(bp)	GC Content	Coding Genes	Average gene size (bp)	Coding region (bp)	tRNAs	rRNAs	Virulence genes (or predicted ones)	Drug resistance genes
R16-chr	5,453,187	57.4%	5,099	930	4,843,803 (88.83%)	85	25	*mrkABCDFHIJ, ybtAEPQSTUX, irp1/2, fyuA, iutA, wzi*, R16_0237, R16_3744, R16_4062	*oqxAB*, *fosA, bla*_SHV-39_, *bla*_LEN-8_, *aadA,emrE*
pR16-CR-HvKp1	144,829	54.3%	182	603	109,800 (75.82%)	0	0	R16_5486	*bla*_kpc-2_, *bla*_CTX-M-65_, *bla*_SHV-12_, *bla*_TEM-1_, *rmtB*
pR16-CR-HvKp2	110,265	52.9%	111	818	90,906 (82.44%)	0	0	/	/
pR16-CR-HvKp3	109,507	49.6%	118	778	93,447 (85.33%)	2	0	/	/

**Figure 7.**
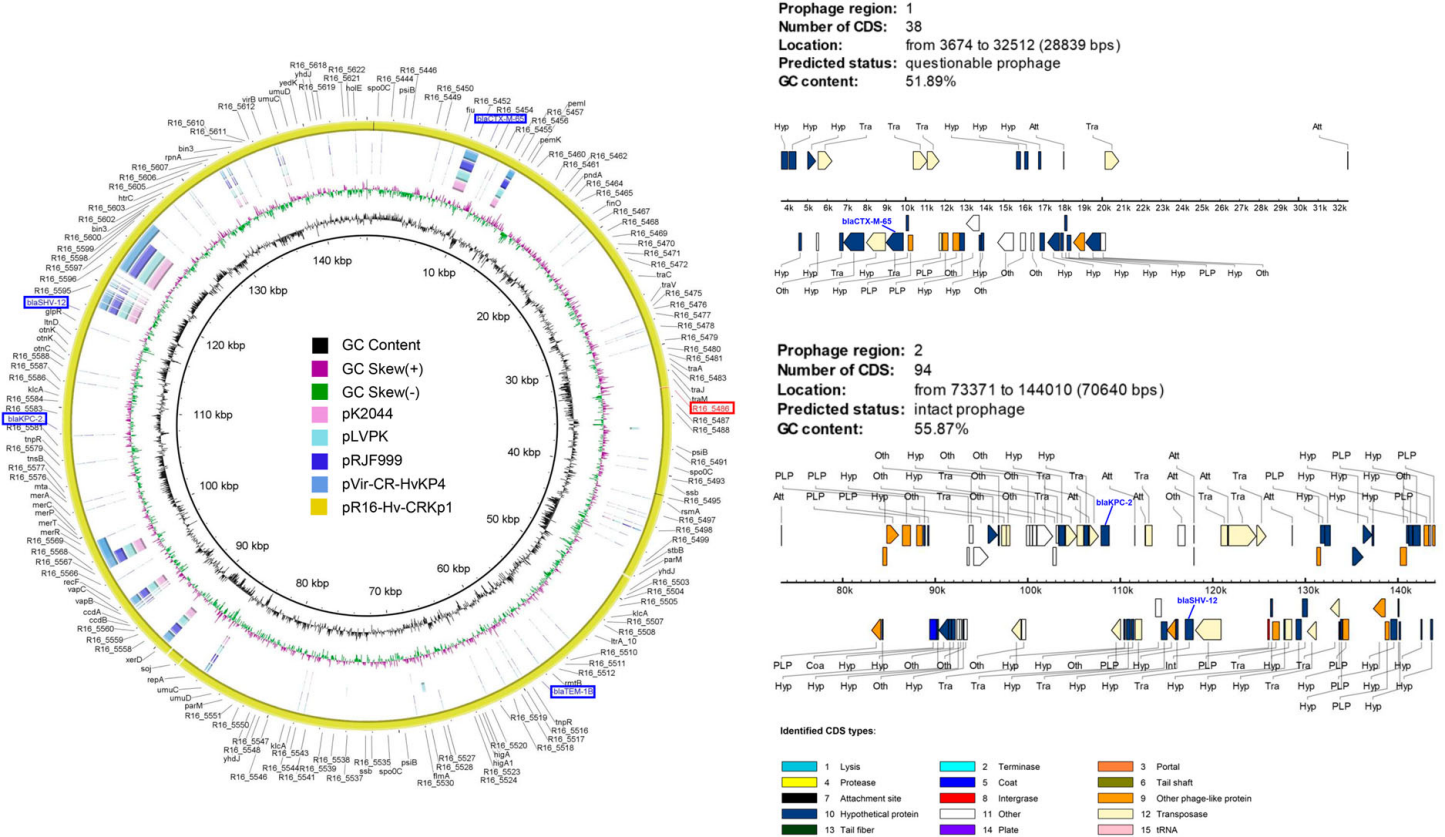
Structure of the plasmid pR16-Hv-CRKp1 and corresponding prophage regions. (A) Structural comparison of the plasmid pR16-Hv-CRKp1 and four known virulence plasmids. Alignment of plasmid pK2044, pLVKP, pRJF999 and pVir-CR-HvKP4 are shown as concentric rings. The outermost show the main coding genes of pR16-Hv-CRKP1. Drug resistance genes are highlighted in blue, and the predicted virulence gene is highlighted in red. (B) Structure of two prophage regions. Different CDS types are shown in different colours. Drug resistance genes are highlighted in blue.

Strain R16 contained three plasmids pR16-Hv-CRKp1, pR16-Hv-CRKp2 and pR16-Hv-CRKp3 (Table 3). We found the pR16-Hv-CRKp1 (141 kb) plasmid to be unique as it harboured the carbapenemase genes *bla*_KPC-2_, beta-lactamase genes *bla*_CTX-M-65_, *bla*_SHV-12_ and *bla*_TEM-1_, aminoglycoside resistance gene *rmtB* together with one predicted virulence gene R16_5486, simultaneously. This plasmid is IncFII type and shared 99% identity with p283747-KPC (GenBank MF168406). To the best of our knowledge, this is the first report on this plasmid. Moreover, not much homologous sequences were found between this plasmid and the known virulent plasmids (pK2044 (GenBank AP006726), pLVKP (GenBank AY378100), pRJF999 (GenBank CP014011) and pVir-CR-HvKP4 (GenBank MF437313)) (Figure 7). Structure analysis revealed two prophages (one intact prophage of 70,640 bp, and one questionable prophage of 28,839 bp) located on pR16-Hv-CRKp1 (Figure 7). The *bla*_KPC-2_ and *bla*_SHV-12_ genes were integrated to R16 through the intact 70,640 bp prophage, and *bla*_CTX-M-65_ was integrated to R16 through the questionable 288,39 bp prophage, endowing R16 with corresponding drug resistance. The presence of the whole plasmid may simultaneously endow R16 with high virulence due to the predicted virulence gene R16_5486.

Additionally, more detailed analysis of other seven CRKPs was performed using R16 as reference genome (Figure S2). We found that six CRKps (R2, R7, R12, R15, R17 and R18) might also possessed this rare plasmid (genome coverage from >77%, gene coverage >80%), and R7 had the highest homology (genome coverage 98%, gene coverage 100%). The other one CRKp strain R5 shared about 50% coverage of pR16-Hv-CRKp1.

## Discussion

In this study, we describe the epidemiology of CRE in patients who presented with bacterial liver abscesses in China from 2012 to 2016, and report on the emergence of ST11-K47 and ST11-K64 Hv-CRKps. Furthermore, we also characterized the genetic basis of the drug-resistance and virulence of these strains.

Our results reveal that ST11 *K. pneumoniae* is the most prevalent CRKp in liver abscess cases in China. Phylogenic analysis demonstrated that CRKp and CSKp strains were clustered in different nodes, indicating that they evolved separately in distinct clonal groups. Drug resistance gene analysis showed that CRKp strains contained almost all classes of resistance genes and exhibited multidrug resistance. The emergence of such strains will lead to failure in antibacterial drug therapy and subsequent prolongation of the course of illness, resulting in increased difficulties and challenges for the treatment of infections. Earlier studies have shown that multidrug-resistant (MDR) *K. pneumoniae* strains have lower virulence, whereas HvKps are sensitive to most antibiotics [9]. Moreover, hypervirulent and MDR *K. pneumoniae* strains have evolved separately in different clonal groups [23]. However, our virulence assay results showed that CRE isolates, R16, R7, R17, R2, R12, R18 and R5, not only had higher virulence levels, but also contained almost all types of antibiotic resistance genes (listed in Figure 3). Unlike the Hv-CSKps that belonged to serotype K1, K2 or K57, these Hv-CRKps are K47 and K64, which has rarely been reported before. As previously reported, ST11 CRKps have been detected widely and ST11 Hv-CRKps have also been described. However, ST11 Hv-CRKps with capsular serotype K47 and K64 have rarely been reported. Until now, only two ST11-K47 Hv-CRKp have been reported and no ST11-K64 ones. All the five K47 CRKp strains showed high virulence, thus we inferred that K47 might also be a serotype related to high virulence. The emergence of ST-K47 and ST-K64 Hv-CRKps will undoubtedly present serious challenges in the management of patients infected with these strains due to its simultaneous possession of resistance and virulence genes.

Comparative analysis of virulence genes between CRKp and CSKp strains showed that the four CSKp ST23 strains (SR6, S1, S2, S8) harboured almost all virulence genes and thus exhibited higher virulence, further demonstrating that ST23 clonal group members are highly virulent. Moreover, we analyzed *Klebsiella pneumoniae* strains (*n* = 206) with complete genomes from NCBI. There were 24 ST11 *K. pneumoniae* strains and only two (strain 911021 and 721005) of them lacked carbapenems resistant genes. Additionally, we conducted a phylogenetic analysis and virulence/antimicrobial resistance gene distribution analysis of these two strains together with our eight ST11 CRKps (Figure S3). We found that our eight ST11 CRKps and these two CSKps were separated into two clusters, so we speculated that the ST11 CRKps and CSKps evolved in different directions, yet more work needed be done.

We also found out that *K. pneumoniae s*trains possessing CPS regulator genes (*rmpA* or *rmpA2*), salmochelin (*iroBCDN*) and aerocin (*iucABCD*) siderophore gene clusters, had higher virulence levels as tested in NKA and *G. mellonella* infection assays. This was further confirmed by experiments involving another *K. pneumoniae* strain, *K. pneumoniae*1084, a hypermucoviscosity-negative K1 clinical strain, which has been shown to be of low virulence in liver abscess infection model [24]. Our study demonstrated that strain 1084 lost CPS regulator gene *rmpA* and salmochelin and aerocin siderophore genes, unlike the high-virulence strain NTUH-K2044 (Figure 6). This result further verifies the notion that these genes play important roles in the virulence phenotype of *K. pneumoniae*. Therefore, the acquisition of high virulence by ST11-K64 Hv-CRKp R5 is very likely to be related to the acquisition of these virulence genes. However, there were notable contradictions between virulence genotypes and phenotypes in all the five ST11-K47 strains (R16, R2, R17, R2, R12) studied, which have a highly virulent phenotype but fewer virulence genes. Nevertheless, the *wzi* gene and another two virulence genes, yersinibactin and *mrk*, were detected in all the Hv-CRKp strains although some studies indicate that they may not be HvKp specific [22]. Moreover, the neutrophil killing assay and the animal experiment in the present study demonstrated that the virulence of these CRKps is comparable to Hv-CSKp strains and definitive HvKp strain K1088 [9].

Homologous gene analysis against VFDB showed that four predicted virulence genes, R16_0237, R16_3744, R16_4062, R16_5486, were only existent in these CRKp strains compared with our seven CSKps (Figure 6). Moreover, the predicted virulence gene, R16_3744, was also found in Kp1084 and the hypervirulent strain NTUH-K2044 (Figure 6). R16_0237 shared 62.16% sequence identity with VFG035256 (ImpA-like protein); R16_3744 shared 63.83% sequence identity with VFG041224 (*ctsH1*, Hcp family T6SS protein CtsH1); R16_4062 shared 61.54% sequence identity with VFG044365 (*fct*, ferrichrysobactin receptor); and R16_5486 shared 60.61% sequence identity with VFG042772 (*cofT*, colonization factor antigen III). These results imply that these four predicted genes might also play important roles in the virulence of *K. pneumoniae*. In particular, the gene R16_5486 is located on the rare plasmid pR16-Hv-CRKp1, which simultaneously contained the carbapenemase genes *bla*_KPC-2_, ESBL genes *bla*_CTX-M-65_, *bla*_SHV-12_ and *bla*_TEM-1B_, and an aminoglycoside resistance gene *rmtB*. Additionally, other Hv-CRKp strains also showed high coverage of pR16-Hv-CRKp1 except for R5 (∼50% coverage), which might be the result of genomic recombination (Figure S2). To the best of our knowledge, this is the first report on this particular plasmid. We speculate that the acquisition of this plasmid might endow these strains with multidrug resistance and high virulence capacity at the same time. In contrast, two CSKps (S1 and S2) strains had a low virulent phenotype but harboured more virulence genes. In our opinion, this phenomenon occurs for multiple reasons. First, the presence of a gene is not necessarily indicative of its importance. The expression of genes is affected by multiple factors, such as gene context, interaction between different genes, antagonistic effects, and so on [25,26]. Moreover, SNPs or DNA modifications might also affect gene expression and function [27]. Thus, more work needs to be done in this area.

Noticeably, we found that some strains showed high virulence in NKA and *G. mellonella* infection model but were negative for the string test (ST11-K47: R2, R7, R12, R16, and R17; ST11-K64, R5, R18; ST1764-K64, S7; ST23-K1, S1 and S2; Figure 6 and Table 4), which indicates that the string test was not sensitive enough to detect the virulence of *K. pneumoniae*. Currently, there is still no consensus definition for HvKp and because of this, studies so far have used various criteria (e.g. string test and/or patients’ clinical features) to define these strains. The string test, which has been used widely to identify HvKp strains, has been shown to have suboptimal identification accuracy despite its handleability, particularly in low-prevalence areas [28]. Clinical criteria are also problematic as the patient's profile is determined not only by the infected strain but also the patient's immune-competence and primary diseases. Moreover, notable variable consistency between the two criteria in HvKp infection has been reported from 51% to 98% [29–32]. Consequently, it is difficult to make comparison between different studies as a strain defined as HvKp in one study may not qualify in another study. In this study, we used comprehensive tests combination including string test, human neutrophil killing assay and *G. mellonella* model to define the virulence of HvKP and the results of the latter two were concordant with each other. Nevertheless, an international consensus definition of HvKp is still needed.

**Table 4.** Characterization of CRE group and comparative CSE group isolates in this study.

Isolate no.	Species	ST	Serotype	Virulence*	MIC (mg/L)			
					Imipenem	Meropenem	Ertapenem	Colistin
Carbapenem-resistant group (*n* = 10)								
R16	*K. pneumoniae*	ST11	K47	H	>32	>16	>4	≤1
R7	*K. pneumoniae*	ST11	K47	H	>32	>16	>4	≤1
R17	*K. pneumoniae*	ST11	K47	H	>32	>16	>4	≤1
R2	*K. pneumoniae*	ST11	K47	H	>32	>16	>4	≤1
R12	*K. pneumoniae*	ST11	K47	H	>32	>16	>4	≤1
R5	*K. pneumoniae*	ST11	K64	H	>32	>16	>4	≤1
R15	*K. pneumoniae*	ST11	K64	M	>32	>16	>4	≤1
R18	*K. pneumoniae*	ST11	K64	H	>32	>16	>4	≤1
R1	*E. coli*	ST88	/	L	≤0.5	1	>4	≤1
R14	*E. cloacae*	ST613	/	L	2	1	4	>4
Carbapenem-susceptible group (*n* = 12, for comparative purpose)								
S7	*K. pneumoniae*	ST1764	K64	H	1	≤0.12	≤0.06	≤1
S3	*K. pneumoniae*	ST412	K57	H	1	≤0.12	≤0.06	≤1
SR3	*K. pneumoniae*	ST65	K2	H	≤0.5	≤0.12	≤0.06	≤1
S8	*K. pneumoniae*	ST23	K1	H	1	≤0.12	≤0.06	≤1
SR6	*K. pneumoniae*	ST23	K1	H	≤0.5	≤0.12	0.12	≤1
S2	*K. pneumoniae*	ST23	K1	M	≤0.5	≤0.12	≤0.06	≤1
S1	*K. pneumoniae*	ST23	K1	M	1	≤0.12	≤0.06	≤1
S4	*E. coli*	ST131	/	H	≤0.5	≤0.12	≤0.06	≤1
S5	*E. coli*	ST131	/	H	≤0.5	≤0.12	0.12	≤1
SR8	*E. coli*	ST38	/	H	≤0.5	≤0.12	0.5	≤1
SR13	*E. coli*	ST2003	/	H	≤0.5	≤0.12	0.25	≤1
SR4	*E. coli*	ST648	/	L	≤0.5	≤0.12	0.12	2

*: H: high; M: medium; L: low.

Study limitations include possible selection bias as the geographic distribution of isolates from participating hospitals were not representative enough, with some remote regions poorly represented.

In summary, this study characterized the emergence of rare ST11-K47 and ST11-K64 Hv-CRKps, and clarified the genetic basis of drug-resistance and virulence phenotypes in CRE/CSE strains, which will provide important insights into the control and treatment of infections involving CRE or even Hv-CRE. More work is needed to understand the detailed pathogenic mechanisms and transmission dynamics of CRE/Hv-CRE strains.

## Contributors

QWY, XMJ, MLZ, WHY, HZ, and YCX conceived and designed the experiments, performed the experiments, analyzed the data, and wrote the paper. Other authors provided the isolates used and approved the final version of the manuscript.

## Supplementary Material

Supplemental Material
