# Supplementary material for: Emergence of ST11-K47 and ST11-K64 hypervirulent carbapenem-resistant Klebsiella pneumoniae in bacterial liver abscesses from China: a molecular, biological, and epidemiological study
Source: Emerg Microbes Infect. 2020 Feb 9;9(1):320–31. doi: 10.1080/22221751.2020.1721334 (PMC7034084; doi:10.1080/22221751.2020.1721334)
Supplement: Supplemental Material [file TEMI_A_1721334_SM9675.zip › Supplementary methods detail S3-20190929.docx]

**Emergence of ST11-K47 and ST11-K64 hypervirulent carbapenem-resistant *Klebsiella pneumoniae* in bacterial liver abscesses from China: a molecular, biological, and epidemiological study**

Qiwen Yang^†*1,2^, Xinmiao Jia^†3^, Meng-Lan Zhou^†,1,2,4^, Hui Zhang^1,2^, Wenhang Yang^1,2^, Yingchun Xu^*1,2^.

^1^Department of Clinical Laboratory, Peking Union Medical College Hospital, Peking Union Medical College, Chinese Academy of Medical Sciences, Beijing 100730, China

^2^Beijing Key Laboratory for Mechanisms Research and Precision Diagnosis of Invasive Fungal Diseases, Beijing, China

^3^Central Research Laboratory, Peking Union Medical College Hospital, Peking Union Medical College, Chinese Academy of Medical Sciences, Beijing, 100730, China

^4^Graduate School, Peking Union Medical College, Chinese Academy of Medical Sciences, Beijing, China

^#^Yang Q, Jia X and Zhou M contributed equally to this study.

**Corresponding Author:**

Prof Qiwen Yang

Email: yangqiwen81@vip.163.com

**and**

Prof Yingchun Xu

Email: xycpumch@139.com

**Supplementary Material S3**

**Methods**

**Virulence assay using the neutrophil killing assay**

Briefly, neutrophils were prepared from healthy volunteers’ blood with written informed consent. An inoculation of 1 × 10^6^ neutrophils with 1 × 10^6^ colony-forming units (CFU) of opsonized *K pneumoniae* were incubated together for 1h before diluting and plating on blood agar for colony count. A previously published hypervirulent *Klebsiella pneumoniae* strain 1088, a gift from Dr. Zhang R was used as the positive control [1]. A classic *K. pneumoniae* strain QD110 identified in this study was added as a negative control. Virulence level was reflected by survival rate as the percentage of CFUs recorded after neutrophil treatment compared with the control: ≥80% referred to high level virulence (HV); ≥50% and <80% referred to middle level virulence (MV); ＜50% referred to low level virulence (LV). Each strain was tested four times and an average survival rate was calculated.

**Virulence assay using *Galleria mellonella* infection model**

Overnight cultures of strains were washed with phosphate-buffered saline (PBS) and further adjusted with PBS to concentrations of 1 × 10^8^ CFU/mL, 1 × 10^7^CFU/mL, 1 × 10^6^CFU/mL, and 1 × 10^5^CFU/mL. We infected the *G. mellonella* with different concentrations of bacteria as previously described,[1] and recorded the survival rate of *G. mellonella*. Strains 1088 and QD110 were used as positive and negative controls, respectively. All experiments were performed in triplicate. Strains’ virulence levels were classified based on the survival rates of *G. mellonella* with 1 × 10^6^ CFU bacteria inoculum at 12h: <= 20% referred to HV; >20% and <=50% referred to MV; >50% referred to LV.

**Genomic DNA extraction, sequencing, assembly and annotation**

Whole-genome sequencing of one ST11-K47 Hv-CRKp strain R16 was further implemented using Pacific Biosciences Sequel System (Pacific Biosciences, Menlo Park, CA, USA). De novo assembly of the genome was performed using HGAP3 within the SMRT Link v5.0.0. Gap closing was completed by PBJelly,[2] and circularization was achieved by manual comparison and removal of regions of overlap. The final genome was further confirmed by remapping of Illuminareads using BWA 0.5.9 and Pilon v.1.13.[3] Gene prediction and annotation of assembled sequences were performed via Prokka.[4] Sequencing reads have been deposited in National Center for Biotechnology Information (NCBI) Sequence Read Archive (SRA) database with SRP141269.

**Virulence genes, antimicrobial resistance genes and homologous genes analysis**

FASTA sequences of virulence and antimicrobial resistance genes were used to search for homologous genes based on the annotated genes in four reference genomes (NTUH-K2044, Kp1084, JM45 and HS11286) using BLAST with 50% coverage and 90% identity. Homologous genes were identified through Inparanoid and Multiparanoid (http://inparanoid.sbc.su.se/cgi-bin/index.cgi).

1. Gu D, Dong N, Zheng Z, et al. A fatal outbreak of ST11 carbapenem-resistant hypervirulent Klebsiella pneumoniae in a Chinese hospital: a molecular epidemiological study. Lancet Infect Dis **2018**; 18(1): 37-46.

2. English AC, Richards S, Han Y, et al. Mind the gap: upgrading genomes with Pacific Biosciences RS long-read sequencing technology. PLoS One **2012**; 7(11): e47768.

3. Walker BJ, Abeel T, Shea T, et al. Pilon: an integrated tool for comprehensive microbial variant detection and genome assembly improvement. PLoS One **2014**; 9(11): e112963.

4. Seemann T. Prokka: rapid prokaryotic genome annotation. Bioinformatics **2014**; 30(14): 2068-9.
