# Supplementary material for: Emergence of ST11-K47 and ST11-K64 hypervirulent carbapenem-resistant Klebsiella pneumoniae in bacterial liver abscesses from China: a molecular, biological, and epidemiological study
Source: Emerg Microbes Infect. 2020 Feb 9;9(1):320–31. doi: 10.1080/22221751.2020.1721334 (PMC7034084; doi:10.1080/22221751.2020.1721334)
Supplement: Supplemental Material [file TEMI_A_1721334_SM9675.zip › Supplementary Table S4-20190929.docx]

**Supplementary Table S4. Clinical information of selective CRE and CSE isolates for comparative genomic analysis in this study.**

| **Sample ID** | **Hospital** | **Sample Type** | **Species^b^** | **Department^c^** | **Sex** | **Age** | **Past history** | | **Treatment process** | | | **Evaluation of therapeutic prognosis** | | |
| --- | --- | --- | --- | --- | --- | --- | --- | --- | --- | --- | --- | --- | --- | --- |
|  |  |  |  |  |  |  | **Inpatient history within 90 days before infection** | **Broad spectrum antibiotic usage within 90 days before infection** | **Antibiotics** | **Course of treatment (day)** | **Surgical intervention^d^** | **Clinical assessment of 72 hours after the antibacterials treatment** | **Bacteriological assessment of 72 hours after the antibacterials treatment** | **Final prognosis** |
| R16 | PU | Liver abscess | kpn | Emergency department | Male | 26 | No | Yes | Ertapenem | 5 | Yes | Remission | Assumed clearance^e^ | Improved and discharged |
| R7 | CY | Liver abscess | kpn | Surgery | Male | 62 | No | No | Cefoxitin | 8 | Yes | Remission | Assumed clearance | Improved and discharged |
| R17 | ZJ | Liver abscess | kpn | Surgery | Male | 23 | No | No | Cefotaxime/Sulbactam | 12 | Yes | Remission | Assumed clearance | Improved and discharged |
| R2 | JZ | Liver abscess | kpn | Surgery | Male | 48 | Yes | No | Tigecycline, Ertapenem | 5, 5 | Yes | Remission | Assumed clearance | Improved and discharged |
| R12 | CY | Liver abscess | kpn | SICU | Male | 64 | No | No | Vancomycin | 20 | Yes | Inefficiency or deterioration | Assumed not-clearance | Death |
| R15 | PU | Liver abscess | kpn | ICU | Male | 62 | Yes | Yes | Tigecycline, Cefoperazone/Sulbactam, Piperacillin/Sulbactam | 4, 3, 4 | Yes | Inefficiency or deterioration | Pathogen persists | Not improved and discharged |
| R18 | ZJ | Liver abscess | kpn | Surgery | Male | 23 | No | No | Piperacillin/Tazobactam, Cefuroxime | 12, 1 | Yes | Remission | Assumed clearance | Improved and discharged |
| R5 | YF | Liver abscess | kpn | Surgery | Male | 85 | No | No | Piperacillin/Tazobactam, Meropenem | 19 | Yes | Inefficiency or deterioration | Assumed not-clearance | Not improved and discharged |
| R1 | JZ | Liver abscess | eco | Surgery | Female | 73 | No | No | Tigecycline, Cefoperazone/Sulbactam | 5, 5 | Yes | Remission | Assumed clearance | Improved and discharged |
| R14 | PU | Liver abscess | ecl | Infectious disease department | Female | 67 | Yes | Yes | Vancomycin, Minocycline, Ceftazidime, Metronidazole, Tigecycline, Cefepime | 21, 21, 24, 20, 8, 8 | Yes | Inefficiency or deterioration | Pathogen persists | Not improved and discharged |
| S7 | SD | Liver abscess | kpn | ICU | Female | 62 | Yes | No | Imipenem, Cefoperazone/Sulbactam | 5 | Yes | Remission | Pathogen persists | Not improved and discharged |
| S3 | YF | Liver abscess | kpn | Infectious disease department | Male | 39 | No | No | Imipenem | 11 | Yes | Remission | Assumed not-clearance | Improved and discharged |
| SR3 | JZ | Liver abscess | kpn | Surgery | Male | 51 | No | No | Imipenem | 3 | Yes | Remission | Assumed clearance | Improved and discharged |
| S8 | ZJ | Liver abscess | kpn | SICU | Male | 62 | Yes | No | Pantoprazole, Imipenem | 3, 9 | Yes | Remission | Assumed clearance | Improved and discharged |
| S2 | JZ | Liver abscess | kpn | Surgery | Male | 60 | No | No | Imipenem | 3 | Yes | Remission | Assumed clearance | Improved and discharged |
| S1 | JZ | Liver abscess | kpn | Surgery | Male | 38 | No | No | Imipenem | 3 | Yes | Remission | Assumed clearance | Improved and discharged |
| S4 | CQ | Liver abscess | eco | Surgery | Male | 56 | Yes | Yes | Imipenem, Cefoperazone/Sulbactam | 5, 5 | Yes | Remission | Pathogen clearance | Improved and discharged |
| S5 | YF | Liver abscess | eco | Surgery | Female | 67 | No | No | Imipenem | 5 | Yes | Remission | Pathogen persists | Improved and discharged |
| SR8 | GZ | Liver abscess | eco | Organ transplantation department | Male | 39 | Yes | No | Piperacillin/Tazobactam, Panipenem, Fluconazole, Meropenem, Vancomycin, Caspofugin, Imipenem | 4, 20, 20, 6, 12, 5, 12 | Yes | Inefficiency or deterioration | Pathogen persists | Improved and discharged |
| SR4 | SD | Liver abscess | eco | Surgery | Female | 55 | No | No | Imipenem, Cefoperazone/Sulbactam | 5, 5 | Yes | Remission | Assumed clearance | Improved and discharged |
| SR13 | CY | Liver abscess | eco | Surgery | Female | 52 | Yes | Yes | Cefoxitin, Cefoperazone/Sulbactam | 1, 3 | Yes | Remission | Assumed clearance | Improved and discharged |
| SR6 | ZS | Liver abscess | kpn | Surgery | Female | 45 | No | No | Imipenem | 5 | Yes | Remission | Assumed clearance | Improved and discharged |

| Notes: | ^a^PU, Peking Union Medical College Hospital; CY, Beijing Chao-Yang Hospital; ZJ, The First Affiliated Hospital of Medical School of Zhejiang University; JZ, The General Hospital of People’s Liberation Army(301 hospital); YF, Sir Run Run Shaw Hospital; SD, Shangdong Provincial Hospital; CQ, The First Affiliated Hospital of Chongqing Medical University; GZ, The First Affiliated Hospital,Sun Yat-sen University. |
| --- | --- |
|  | ^b^kpn, *Klebsiella pneumoniae*; eco, *Escherichia coli*; ecl, *Enterobacter cloacae*. |
|  | ^c^ICU, intensive care unit; SICU, surgical intensive care unit. |
|  | ^d^Surgical intervention includes liver abscess puncture drainage and/or laparotomy and/or necrotic infection tissue resection and/or T-tube drainage and/or peri hepatic puncture drainage and/or hemofiltration and/or percutaneous drainage of biliary tract and/or choledocholithotomy and/or intrahepatic biliary duct drainage. |
|  | ^e^Assumed clearance: symptomatic signs disappear but no bacteriological specimens; Assumed not-clearance: symptomatic signs exist but no bacteriological specimens. |
