# Supplementary material for: Emergence of ST11-K47 and ST11-K64 hypervirulent carbapenem-resistant Klebsiella pneumoniae in bacterial liver abscesses from China: a molecular, biological, and epidemiological study
Source: Emerg Microbes Infect. 2020 Feb 9;9(1):320–31. doi: 10.1080/22221751.2020.1721334 (PMC7034084; doi:10.1080/22221751.2020.1721334)
Supplement: Supplemental Material [file TEMI_A_1721334_SM9675.zip › Supplementary Table S5-20190929.docx]

Supplementary Table S5. Risk factor analysis between CRE and CSE group.

| **Variables** | **CRE (n=10)** | **CSE (n=12)** | **p** | **Methods** |
| --- | --- | --- | --- | --- |
| Sex (male/female) | 8/2 | 7/3 | 0.381 | fisher's exact test |
| Age (years) | 52.90±22.86 | 52.00±9.83 | 0.465 | Mann-Whitney test |
| Patient source (outpatient/emergency/other hospitals/nursing homes) | 2/3/1/4 | 3/5/2/2 | 0.734 | fisher's exact test |
| Pulmonary diseases (Yes/No) | 0/10 | 1/11 | 1.000 | fisher's exact test |
| Tumor (Yes/No) | 4/6 | 1/11 | 0.135 | fisher's exact test |
| Liver diseases (Yes/No) | 5/5 | 6/6 | 1.000 | fisher's exact test |
| Heart Diseases (Yes/No) | 2/8 | 0/12 | 0.195 | fisher's exact test |
| Nervous system disease (Yes/No) | 1/9 | 0/12 | 0.455 | fisher's exact test |
| Kidney disease (Yes/No) | 0/10 | 2/10 | 0.481 | fisher's exact test |
| Diabetes (Yes/No) | 0/10 | 4/8 | 0.096 | fisher's exact test |
| Injecting drug use (Yes/No) | 0/10 | 0/12 | 1.000 | fisher's exact test |
| Drinking history (Yes/No) | 2/8 | 2/10 | 1.000 | fisher's exact test |
| Smoking history (Yes/No) | 2/8 | 2/10 | 1.000 | fisher's exact test |
| Neutropenia (Yes/No) | 0/10 | 0/12 | 1.000 | fisher's exact test |
| Splenectomy (Yes/No) | 0/10 | 0/12 | 1.000 | fisher's exact test |
| Recent surgery (Yes/No) | 2/8 | 5/7 | 0.381 | fisher's exact test |
| Hormones used in recent 3 months (Yes/No) | 0/10 | 0/12 | 1.000 | fisher's exact test |
| Immunosuppressor used in recent 3 months (Yes/No) | 0/10 | 1/11 | 1.000 | fisher's exact test |
| Inpatient history within 90 days before infection (Yes/No) | 3/7 | 5/7 | 0.675 | fisher's exact test |
| Antibiotic usage within 90 days before infection (Yes/No) | 3/7 | 3/9 | 1.000 | fisher's exact test |

CRE: carbapenem-resistant *Enterobacteriaceae*; CSE: carbapenem-susceptible *Enterobacteriaceae*
