# Supplementary figures and images for: Emergence of ST11-K47 and ST11-K64 hypervirulent carbapenem-resistant Klebsiella pneumoniae in bacterial liver abscesses from China: a molecular, biological, and epidemiological study
Source: Emerg Microbes Infect. 2020 Feb 9;9(1):320–31. doi: 10.1080/22221751.2020.1721334 (PMC7034084; doi:10.1080/22221751.2020.1721334)

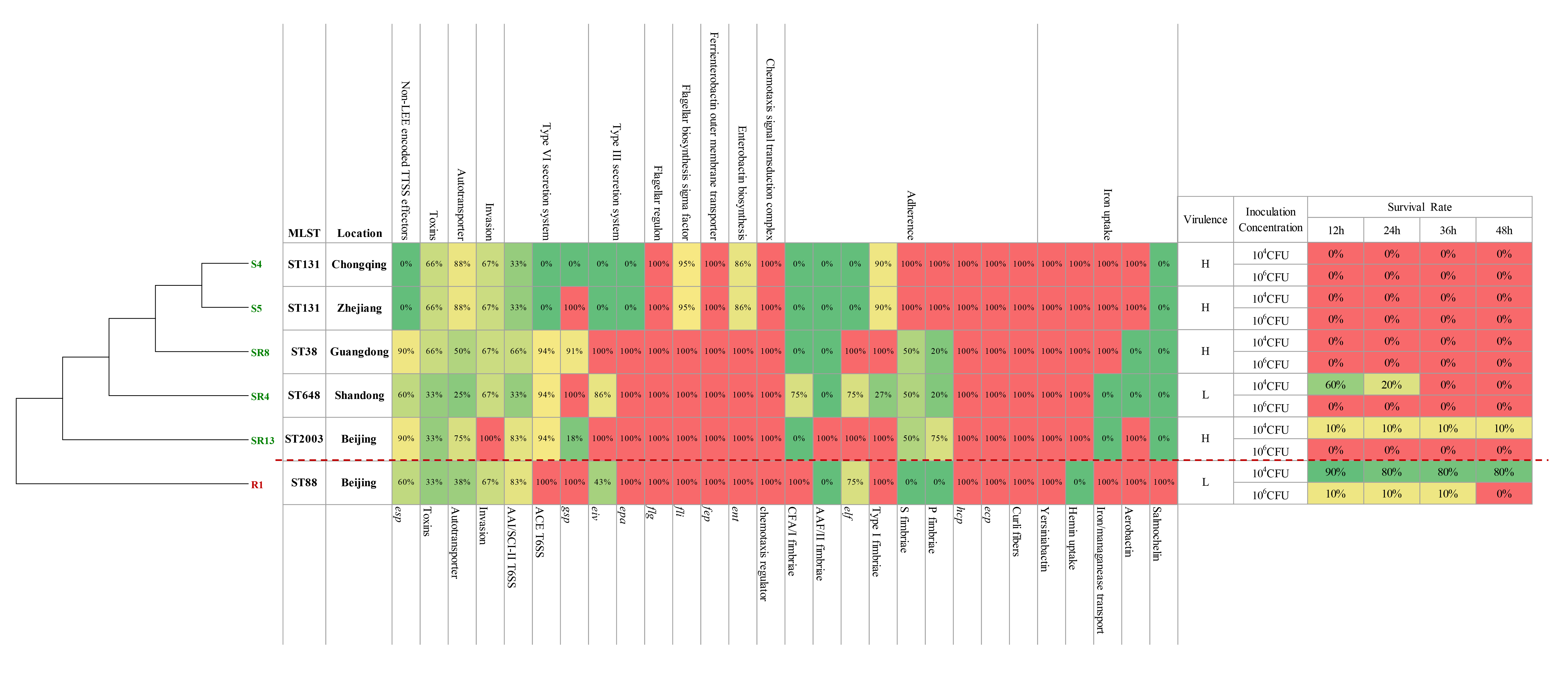

Supplement: Supplemental Material [file TEMI_A_1721334_SM9675.zip › Figure S1.tif]

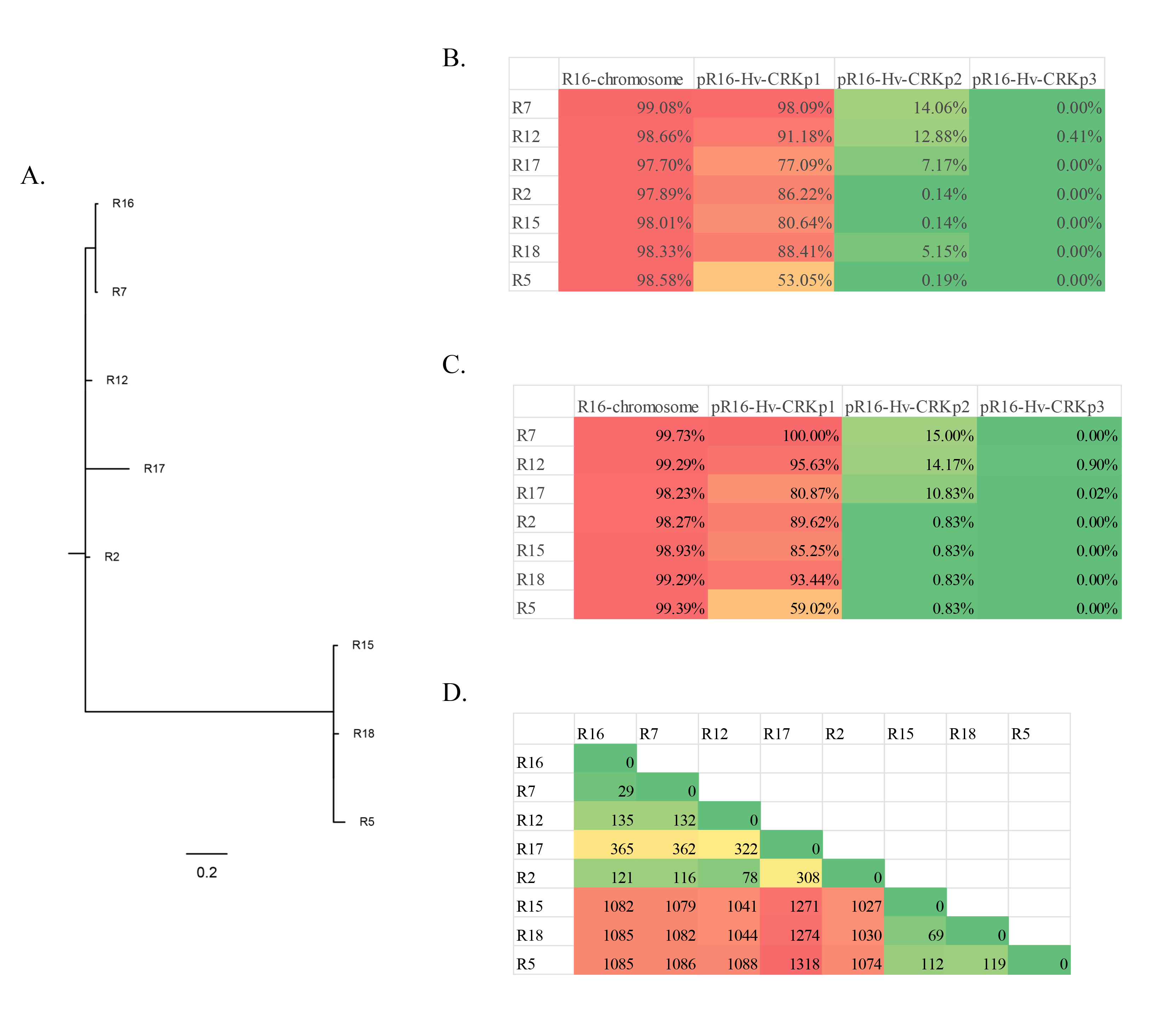

Supplement: Supplemental Material [file TEMI_A_1721334_SM9675.zip › Figure S2.tif]

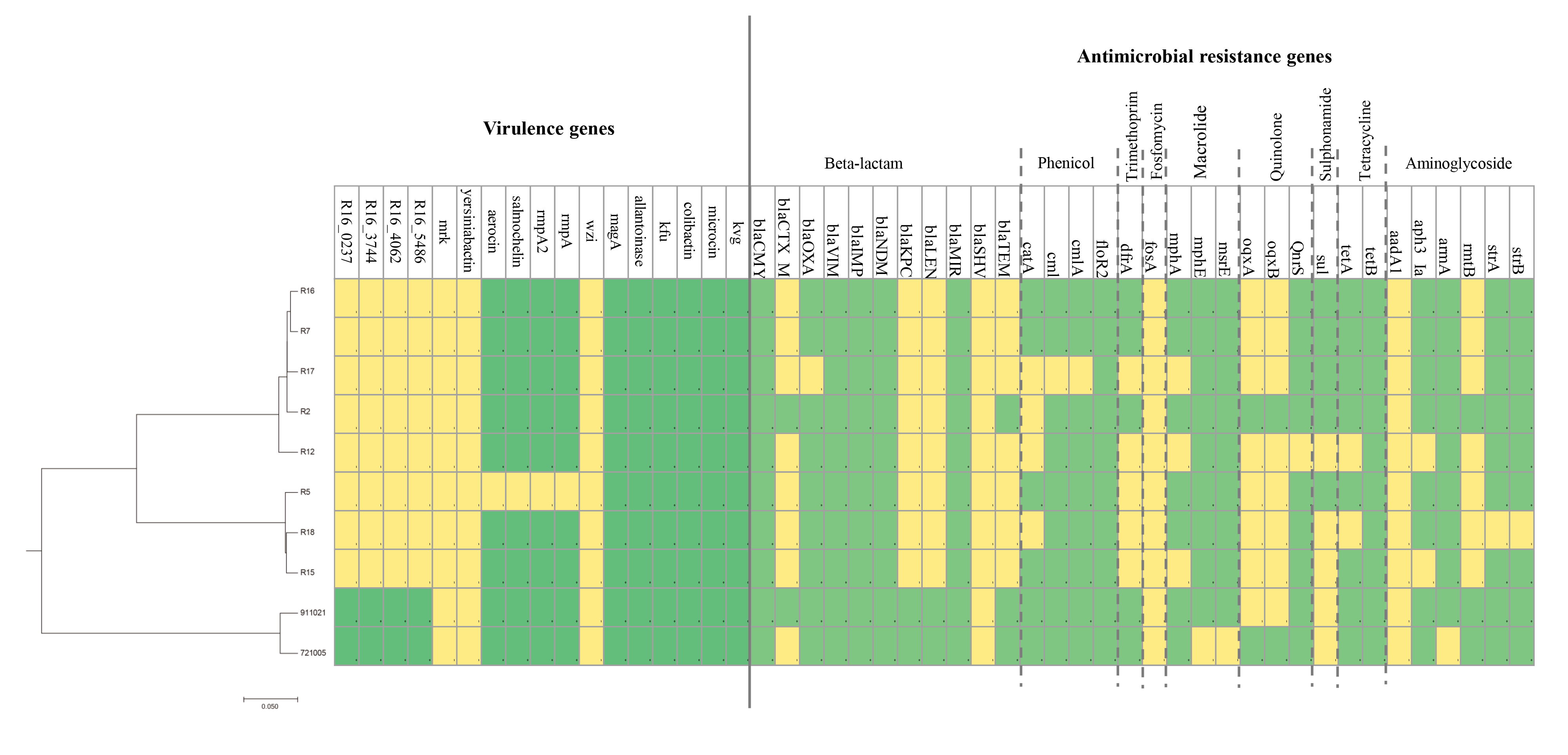

Supplement: Supplemental Material [file TEMI_A_1721334_SM9675.zip › Figure S3 new.tif]
